# Functional Characterization of Dihydroflavonol-4-Reductase in Anthocyanin Biosynthesis of Purple Sweet Potato Underlies the Direct Evidence of Anthocyanins Function against Abiotic Stresses

**DOI:** 10.1371/journal.pone.0078484

**Published:** 2013-11-04

**Authors:** Hongxia Wang, Weijuan Fan, Hong Li, Jun Yang, Jirong Huang, Peng Zhang

**Affiliations:** 1 National Key Laboratory of Plant Molecular Genetics and National Center for Plant Gene Research (Shanghai), Institute of Plant Physiology and Ecology, Shanghai Institutes for Biological Sciences, Chinese Academy of Science, Shanghai, China; 2 Shanghai Chenshan Plant Science Research Center, Chinese Academy of Science, Chenshan Botanical Garden, Shanghai, China; Kansas State University, United States of America

## Abstract

Dihydroflavonol-4-reductase (DFR) is a key enzyme in the catalysis of the stereospecific reduction of dihydroflavonols to leucoanthocyanidins in anthocyanin biosynthesis. In the purple sweet potato (*Ipomoea batatas* Lam.) cv. Ayamurasaki, expression of the *IbDFR* gene was strongly associated with anthocyanin accumulation in leaves, stems and roots. Overexpression of the *IbDFR* in Arabidopsis *tt3* mutants fully complemented the pigmentation phenotype of the seed coat, cotyledon and hypocotyl. Downregulation of *IbDFR* expression in transgenic sweet potato (DFRi) using an RNAi approach dramatically reduced anthocyanin accumulation in young leaves, stems and storage roots. In contrast, the increase of flavonols quercetin-3-O-hexose-hexoside and quercetin-3-O-glucoside in the leaves and roots of DFRi plants is significant. Therefore, the metabolic pathway channeled greater flavonol influx in the DFRi plants when their anthocyanin and proanthocyanidin accumulation were decreased. These plants also displayed reduced antioxidant capacity compared to the wild type. After 24 h of cold treatment and 2 h recovery, the wild-type plants were almost fully restored to the initial phenotype compared to the slower recovery of DFRi plants, in which the levels of electrolyte leakage and hydrogen peroxide accumulation were dramatically increased. These results provide direct evidence of anthocyanins function in the protection against oxidative stress in the sweet potato. The molecular characterization of the *IbDFR* gene in the sweet potato not only confirms its important roles in flavonoid metabolism but also supports the protective function of anthocyanins of enhanced scavenging of reactive oxygen radicals in plants under stressful conditions.

## Introduction

Anthocyanins, a class of flavonoids that is responsible for the colors in fruits and most flowers of higher plants, are major water-soluble pigments [1], [2]. They have been reported to exhibit important physiological functions, such as antioxidative [3], [4], antimutagenic [5] and anticancer activities [6], [7]. Anthocyanin biosynthesis has been well characterized in several plants, such as Arabidopsis (*Arabidopsis thaliana*), maize (*Zea mays*), snapdragon (*Anfirrhinum majus*) and petunia (*Petunia hybrida*) [8], [9], [10], [11]. First, the upstream phenylpropanoid pathway converts the substrate L-phenylalanine to 4-coumarate CoA using phenylalanine ammonialyase (PAL), cinnamate 4-hydroxylase (C4H) and 4-coumarate CoA ligase (4CL). Next, 4-coumarate CoA is catalyzed by chalcone synthase (CHS), chalcone isomerase (CHI) and flavanone 3-hydroxylase (F3H) to form dihydroflavonol. Finally, dihydroflavonol 4-reductase (DFR) catalyzes dihydroflavanones to leucoanthocyanidins, which then are converted to anthocyanidin by anthocyanindin synthase (ANS). Therefore, DFR is a key enzyme in anthocyanin biosynthesis that controls the carbon flux direction. Simplified scheme of the anthocyanin biosynthesis pathway is described in Figure S1.

DFR has also been known to affect the biosynthesis of other flavonoids, e.g. flavonols and proanthocyanidin [12], [13], [14]. The substrate dihydroflavonol of DFR can be catalyzed by FLS to produce flavonols, and the leucoanthocyanidins that result from DFR can subsequently be converted to proanthocyanidin by leucoanthocyanidin reductase (LAR) [15], [16], [17]. These are the two key branches in flavonoid pathways. Therefore, further understanding of DFR function in the regulation of flavonoid biosynthesis in plants is of importance.

DFR uses NADPH as a cofactor to catalyze the reduction of dihydroflavonols to their respective colorless, unstable leucoanthocyanidins, which are common precursors for anthocyanin and proanthocyanidin biosynthesis [10], [18], [19], [20]. Studies have demonstrated that deactivation of the *DFR* gene results in the loss of anthocyanins and proanthocyanidin in mutants of barley and Arabidopsis [21], [22]. In Arabidopsis, *tt3* (*transparent testa*) mutants lacking DFR activities failed to accumulate the brown tannins of proanthocyanidin in their seed coats. In addition, those mutants showed no anthocyanin pigments within the cotyledon or hypocotyl when grown in Murashige and Skoog media with a low nitrogen content unlike wild-type (WT) Arabidopsis seedlings, which had strong red pigmentation [22], [23]. Expression of the maize *A1* gene encoding a DFR under the control of the CaMV 35S promoter in the *tt3* mutants could restore the pigmentation within the cotyledon and seed coat under low-nitrogen conditions [24]. Due to their crucial role in the flavonoid pathway, various DFR genes have been isolated from other species such as grape (*Vitis vinifera*), apple (*Malus domestica*), pear (*Pyrus communis*), sweet orange (*Citrus sinensis*) and petunia (*Petunia hybrida*) [25], [26], [27], [28].

Attention is now being focused on the purple sweet potato (*Ipomoea batatas* Lam.) because of its unique color and its nutritive and health-promoting benefits [29], [30]. Many anthocyanins have been isolated and identified in purple sweet potato [30], [31], [32], [33]. For example, twenty-six anthocyanins were detected and characterized in the aqueous extract of the purple line cell line. These anthocyanins are exclusively cyanidin or peonidin 3-sophoroside-5-glucosides and their acylated derivatives [34]. Many studies have reported that purple sweet potato anthocyanins can protect the rat liver from hepatoxin-induced injury [35] and have the antioxidative ability to scavenge active oxygen radicals [36]. However, due to the unclear genetic background of those materials in those studies (i.e., the sweet potato is a vegetatively propagated allopolyploidy root crop), it is difficult to confirm the pharmaceutical function of sweet potato anthocyanins. Therefore, it is essential to use sweet potato with the same genetic background with or without anthocyanins to study the regulation of anthocyanin biosynthesis and function. Meanwhile, flavonoids as antioxidants also protect plants under stressful conditions, but their biological function has not yet to be confirmed in sweet potato.

In the present study, we isolated the *IbDFR* gene from sweet potato and investigated its expression profiles in various tissues. Downregulation of *IbDFR* expression by RNAi showed inhibited anthocyanin and proanthocyanidin accumulation and increased flavonol influx. The protective function of anthocyanins in sweet potato was also evidenced by enhanced scavenging of reactive oxygen species (ROS) at low temperature.

## Materials and Methods

### Plant materials

The purple-fleshed sweet potato (*Ipomoea batatas* Lam.) cv. Ayamurasaki was used to produce these transgenic plants. *In vitro* shoot cultures were subcultured on MS medium. One-month-old shoots were transplanted into plastic pots containing well-mixed soil (soil:peat:perlite, 1∶1∶1) and grown in a greenhouse (16 h/8 h light/dark cycle, 25°C day/night). Arabidopsis and its mutant were planted in the greenhouse as described previously (16 h/8 h light/dark cycle, 22°C day/night).

### Cloning of the full-length cDNA of *IbDFR*

A cDNA library was constructed from Ayamurasaki roots. Colonies were transferred onto Hybond-N+ nylon membranes, then denatured and fixed. The polymerase chain reaction (PCR) fragment previously isolated from common morning glory was used as a probe. The membranes were prehybridized for 1 h at 42°C before the probe was added and hybridized in the manufacturer’s hybridization buffer at 42°C overnight. Labeling of the probe was carried out according to the manufacturer’s instructions using the DIG-High Prime DNA Labeling and DetectionStarter Kit II (Roche, Mannheim, Germany). Positively hybridized plaques were isolated and sequenced. Total RNA was extracted from sweet potato roots using an RNAprep Pure Plant kit (Tiangen, Beijing, China), and then reverse-transcribed in a final reaction volume of 20 µL using M-MLV Reverse Transcriptase RNaseH (Toyobo, Osaka, Japan) and oligo(dT)17 primer. The first-strand cDNA was synthesized at 42°C for 1 h and terminated by heating at 95°C for 10 min. Next, 3′RACE PCR was performed using 50-fold diluted cDNA samples as template. The outer primer was 5′-TACCGTCGTTCCACTAGTGATTT -3′ and the inner primer was 5′-CGCGGATCCTCCACTAGTGATTTCACTATAGG-3′. The PCR product was ligated into the pMD-18T vector (TakaRa Dalian Co. Ltd., China) and sequenced by a commercial sequencing service. The final PCR amplification was conducted using the forward *IbDFR* primer 5′-ATGGTGGACGGTAATCATCC-3′ and the reverse *IbDFR* primer 5′-TCAAGCTTTTAAGGGCACTA-3′ to obtain the full-length sequences. The deduced amino acid sequences were used for multiple alignments and phylogenetic tree analysis. The tree was obtained using the ClustalW analysis program [37].

### Analysis of *IbDFR* expression in purple sweet potato

Real-time quantitative qPCR was conducted to investigate the expression profiles of *IbDFR* in different sweet potato tissues including fibrous roots (Ft, maximum diameter <2 mm), development roots (Dt, 2 mm < maximum diameter < 5 mm), storage roots (St, maximum diameter > 5 mm), stems (Sm), and leaves (Lf). Total RNA extracted from the above samples using a RNAprep Pure Plant kit (Tiangen, Beijing, China) was treated with DNase and reverse transcribed using M-MLV Reverse Transcriptase RNaseH (Toyobo, Osaka, Japan). Gene expression was determined using real-time qPCR with the SYBR green method in a Bio-Rad CFX96 thermocycler (Bio-Rad, USA). The real-time qPCR cycling parameters were initial denaturation at 95°C for 1 min, followed by 40 cycles of 95°C for 20 s, 60°C for 20 s and 72°C for 20 s and a final extension at 72°C for 5 min. The primers qIbDFRF (5′-TTATCGGCTCCTGGTTGGT-3′) and qIbDFRR (5′-TGTCCGCTTTCGGTAGTTC-3′) were used for real-time qPCR to amplify a 119-bp fragment. The *IbDFR* expression data were normalized against the expression levels of an internal control *IbActin* gene (forward primer 256 [5′-CTGGTGTTATGGTTGGGATGG-3′], reverse primer 462 [5′- GGGGTGCCTCGGTAAGAAG-3′]). The *IbActin* gene was designed to amplify a 207-bp fragment. The PCR products were confirmed using agarose gel electrophoresis and sequencing. Quantitation of the gene expression was carried out using the comparative Ct method [38]. Each data point represents the average of three independent experiments.

### Complementation of *IbDFR* expression in Arabidopsis mutants

The open reading frame of *IbDFR* was amplified using the primers IbDFR1F

(5′- CGGGGTACCATGGTGGACGGTAATCATCC-3′; *Kpn*I restriction site underlined) and IbDFR1R (5′-AATGTCGACTCAAGCTTTTAAGGGCACTA-3′; *Sal*I restriction site underlined), and *IbDFR* was subcloned into the vector pCAMBIA1301s containing the constitutive CaMV 35S promoter. *A. thaliana tt3* mutants (DFR; At5g42800) were transformed using *Agrobacterium tumefaciens* strain EHA105. Transformants were selected by growing on 1/2 MS medium containing 25 mg/L hygromycin. T3 homozygote RNA extracted from seedlings was used for reverse transcription PCR (RT-PCR) to confirm expression of the *IbDFR* gene in the transformed mutant lines. The *AtActin* gene was used as an internal reference.

### Construction of a hairpin RNA interference (hpRNAi) expression vector and transformation of sweet potato by *A. tumefaciens*

Two 252-bp fragments from *IbDFR* full-length cDNA sequence were amplified by PCR using the primers sense IbDFRi F, 5′-CGGGGTACCAAGGAAGCATGGAAAGCA-3′ (*Kpn*I restriction site underlined) and R, 5′-CCATCGATAGAAGAGCAGATGAATCT-3′ (*Cla*I restriction site underlined); antisense IbDFRi F, 5′-CCGCTCGAGAGAAGAGCAGATGAATCT-3′ (*Xho*I restriction site underlined) and R, 5′-CGGGATCCAAGGAAGCATGGAAAGCA-3′ (*Bam*HI restriction site underlined). Both amplified fragments were subcloned into the TA cloning vector and then subjected to sequence analysis. The two same sequences were inserted into mediate vector to become inverted repeat sequence. The binary vector pRNAi-DFR harboring the expression cassettes having the inverted repeat hairpin structures were constructed. The coding region of the *IbDFR* gene was used as a target sequence and connected in sense using *Kpn*I and *Cla*I and in antisense using *Bam*HI and *Xho*I in the vector. The hairpin structure was driven under control of the cauliflower mosaic virus (CaMV) 35S promoter and hygromycin phosphotransferase gene (*hpt*) as a selectable marker gene (Figure S2A).

The transformation of sweet potato was previously as described by Yang *et al.* (2011) [39]. Embryogenic calli were induced from the bud tissues and subcultured in MSD medium before being transferred to LCP liquid medium for further multiplication. The embryogenic suspensions were transformed with *A. tumefaciens* strain EHA105 harboring the binary vector pRNAi-DFR. The transformed calli were selected on fresh MSD medium with appropriate antibiotics every week for a period of one month and then finally transferred to plant regeneration medium [40].

### Southern blot analysis of transformed plants

Genomic DNA was isolated from leaves of one-month-old *in vitro* seedlings using the cetyltrimethyl ammonium bromide method for DNA gel blot analysis. Twenty micrograms of genomic DNA was digested with *Eco*RI, fragmented on a 0.8% agarose gel, transferred to an Amersham Hybond N^+^ nylon membrane (GE Healthcare, Life Sciences, Indianapolis, USA) and hybridized. Southern blot analysis of the transformants was performed using the DIG-labeled probes of *hpt*. The probe was prepared using a PCR-DIG Probe Synthesis Kit (Roche Diagnostics, Manheim, Germany).

### Extraction and quantification of anthocyanins

The total anthocyanin content in the WT and transgenic lines was extracted using previously described methods [41]. Briefly, approximately 100 mg of lyophilized young leaves or 500 mg of lyophilized storage roots was extracted twice with 10 mL of deionized water containing 5% formic acid and was vortexed for 2 min. The suspensions were centrifuged at 4,000 rpm for 10 min, and the supernatants were combined and filtered through a 0.20-µm nylon filter. The absorbance at OD530 was measured with a spectrophotometer (DU730UV VIS, Beckman Coulter, USA). The color value was calculated using the following formula: CV  =  0.1×OD530×4×20/l FW, where CV is the color value, four and twenty are the dilution rates and FW is the fresh weight of the tissue in grams. Cyanidin 3-O-sophoroside also be measured at OD530. The total anthocyanin content in the WT and transgenic lines can be quantified as cyanidin 3-O-sophoroside equivalents [42].

### Extraction and quantification of flavonols

The total flavonol content in the WT and transgenic lines was extracted and separated by HPLC mass spectrometry using methods described previously with slight modification [19], [43]. Briefly, approximately 200 mg of lyophilized young leaves or 500 mg of lyophilized storage roots was extracted twice with 5 mL or 7 mL of acetone/water/acetic acid (70:29.5:0.5, v/v/v), respectively, and was vortexed for 2 min. The suspensions were centrifuged at 4,000 rpm for 10 min and the supernatants were combined and filtered through a 0.20-µm nylon filter. The analysis of phenolic compounds was performed on an Agilent HPLC1200-MSD/Q-TOF 6520 system (Agilent, Waldbronn, Germany). The system coupled an electrospray ionization (ESI) source with a dual nebulizers allowing reference mass corrected prior to be monitoring. The data obtained from LC and MSD were processed with the Agilent sourced software Masshunter Qualitative Analysis (Version 3.0); it provides calculation of accurate molecular mass and spectrum figure. In HPLC separation, a column (Agilent ZORBAX Eclipse XDB C18 4.6×50 mm, 1.8 µm) was connected behind an autosampler and washed at flow rate of 0.2 mL/min by the mobile phases consisted of 0.5% (v/v) acetic acid in water (eluent A) and 100% acetonitrile (eluent B) when each injection started with ratio of eluent B to A had changing from 0 min on 5% to 20 min on 30% at the temperature of 35°C. Simultaneously, signals of UV on 280 nm, 320 nm, and 370 nm were reserved. Mass data m/z ranging from 40 to1500 was collected under conditions of fragmentor voltage on 160V and Skimer 65V, RF 770V and Vcap 3500V, paralleling the ESI source dry N2 gas pressure was 40psi at temperature 35°C in a flow rate of per min 9L in negative mode. Quantification of flavonols using external calibration curves of quercetin-3-o-glucoside standards. The flavonol concentration was detected in triplicate.

### Proanthocyanidin extraction and quantification

Quantification of total proanthocyanidin from the WT and transgenic plants was performed using the vanillin-HCl method as described previously [44], [45]. The storage roots were ground in liquid nitrogen and extracted using 5 mL of extraction solution (a 1% (v/v) vanillin solution in methanol and an 8% HCl volumetric mix). Following centrifugation at 2,500 × *g* for 10 min, the residues were re-extracted twice as described above. The pooled supernatants were incubated for 20 min at room temperature. The samples and blanks were read at 500 nm using a UV/Vis spectrophotometer. The concentration of proanthocyanidin was detected in triplicate.

### Total antioxidant capacity analysis

To assay antioxidant activities, anthocyanins were extracted using mature storage roots of greenhouse-grown plants. Total antioxidant capacity in the transgenic plants was determined using ferric reducing ability of plasma (FRAP) methods as described previously with slight modifications [46] using a FRAP reagent kit (Biyuntian, Shanghai, China). Briefly, antioxidants can reduce ferric-tripyridyltriazine (Fe^3+^-TPTZ, colorless) into Fe^2+^-TPTZ (blue) in acid. The absorbance was read at 593 nm and the total antioxidant capacity was calculated according to its absorbance. An external standard curve was made with FeSO_4_·7H_2_O, and the result was the relative antioxidant activities. All determinations were conducted in triplicate.

### Cold treatment ion leakage analysis

For the cold treatment, the one-month-old plants in pots were transferred to a refrigerated growth incubator (Friocell404; MMM Medcenter Einrichtungen GmbH, Gräfelfing, Germany) and maintained at 4°C for 24 h. The plants were then transferred to 25°C storage for recovery. For evaluating cellular damage under cold stress, thirty leaf discs (6-mm diameter) collected from the second leaves of the cold treatment plants were assayed by the measuring of electrolyte leakage through membranes as described by Bowler *et al.* (1991) [47]. The leaf discs were floated on deionized water (10 mL) and the ion leakage was assessed using an ion conductivity meter (FE30; Mettler Toledo, Zurich, Switzerland) over a 3-h period. After 3 h, the samples were boiled for 10 min to release the solutes. Solution conductivity was measured again, and this value was considered 100% ion leakage in calculations of the relative ion leakage at different time points. The treatment was performed in triplicate.

### Qualitative and quantitative analysis of H_2_O_2_

To visualize H_2_O_2_ production, the sweet potato leaves were placed in 3-diaminobenzidine (DAB) solution, pH 3.8. The samples were subsequently incubated for 12 h in a growth chamber and the chlorophyll was removed at 80°C for 2 h in 80% ethanol. The H_2_O_2_ content was assessed according to Sairam and Srivastava [48]. The concentration of H_2_O_2_ was estimated by measuring the spectrum absorbance of the titanium-hydroperoxide complex and using a standard curve plotted with known concentrations of H_2_O_2_.

### Statistical analysis

All data were represented as mean ± SD from at least three independent experiments with three replicates each. Statistical significances of the differences were determined using Student’s *t*-test. Differences between treatments were considered significant when P<0.05 or 0.01 in a two-tailed analysis.

## Result

### Cloning and sequence analysis of *IbDFR*

**Figure 1:**
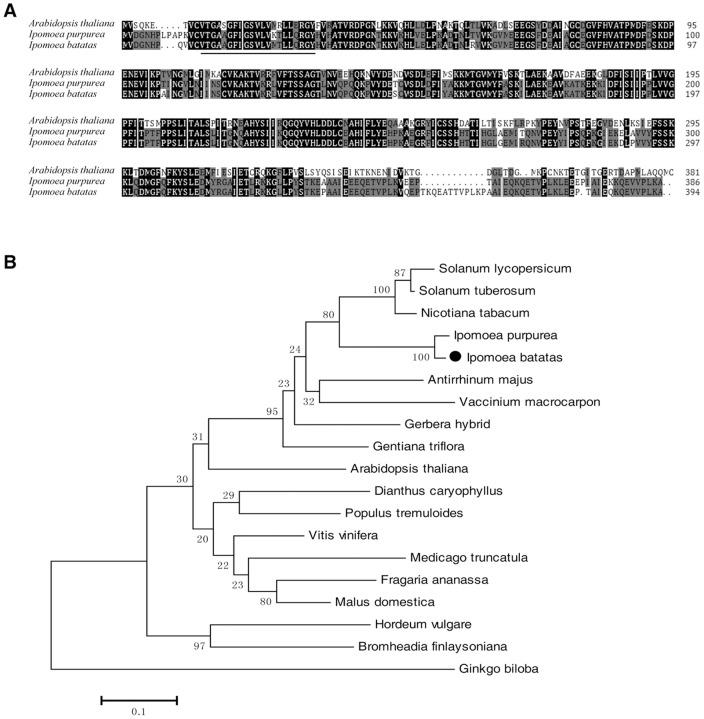
Alignment of amino acid sequences and phylogenetic analysis of the IbDFR protein with other species. (A) Multiple alignment of deduced amino acid sequences of dihydroflavonol-4-reductase (DFR) from sweet potato, *Arabidopsis* and *Ipomoea purpurea*. The black and other shaded boxes show identical and similar amino acids, respectively. The underline indicates the putative NADP binding site. (B) A phylogenetic tree of known plant DFR for anthocyanin biosynthesis. The GenBank accession numbers are: *Arabidopsis thaliana* (*At*DFR, BAD95233), *Fragaria ananassa* (*Fa*DFR, AAC25960), *Malus domestica* (*Md*DFR, AAD26204), *Vitis vinifera* (*Vv*DFR, XP_002281858), *Dianthus caryophyllus* (*Dc*DFR, CAA91924), *Gerbera hybrid* (*Gh*DFR, CAA78930), *Gentiana triflora* (*Gt*DFR, BAA12736), *Ipomoea purpurea* (*Ip*DFR, BAA74700), *Nicotiana tabacum* (*Nt*DFR, ABN80437), *Solanum lycopersicum* (*Sl*DFR, CAA79154), *Vaccinium macrocarpon* (*Vm*DFR, AAL89714), *Hordeum vulgare* (*Hv*DFR, AAB20555), *Bromheadia finlaysoniana* (*Bf*DFR, AAB62873), *Ginkgo biloba* (*Gb*DFR, AAU95082), *Antirrhinum majus* (*Am*DFR, P14721), *Medicago truncatula* (*Mt*DFR1, AAR27014), *Populus tremuloides* (*Pt*DFR, AY147903), *Solanum tuberosum* (*St*DFR, AEN83503). The horizontal scale shows the number of differences per 100 residues derived from Clustal W alignment.

To clone the *IbDFR* gene, a cDNA library constructed from the roots of cv. Ayamurasaki was screened using a cDNA probe from common morning glory (*Ipomoea purpurea*). The positive clones were isolated and sequenced. Several were found to be DFR homologous, and the full sequence of the gene containing the initiation codon (ATG) and the terminator codon (TAA) was obtained (GenBank accession No. EF108570). The 1,185-bp full length of *IbDFR* CDS encodes a protein with 394 amino acids (aa) with a calculated molecular mass of 44.26 KDa and an isoelectric point of 6.477. Amino acid sequence alignments showed that the *IbDFR* gene shared 58.1% and 92.2% identity with *At*DFR and *Ip*DFR, respectively. A putative NADP-binding site (aa 15–35, vtgaagfigswlvktllqrgy) with very high sequence similarity with other DFRs [49] was also present in the N-terminus of the sweet potato DFR (Figure 1A). Phylogenetic tree analysis of DFR amino acid sequences from various species showed that DFR proteins were clustered into three distinct groups (Figure 1B). *Ib*DFR and *At*DFR belong to different branches of the tree.

### Anthocyanin accumulation and expression profiles of *IbDFR* in different organs

**Figure 2:**
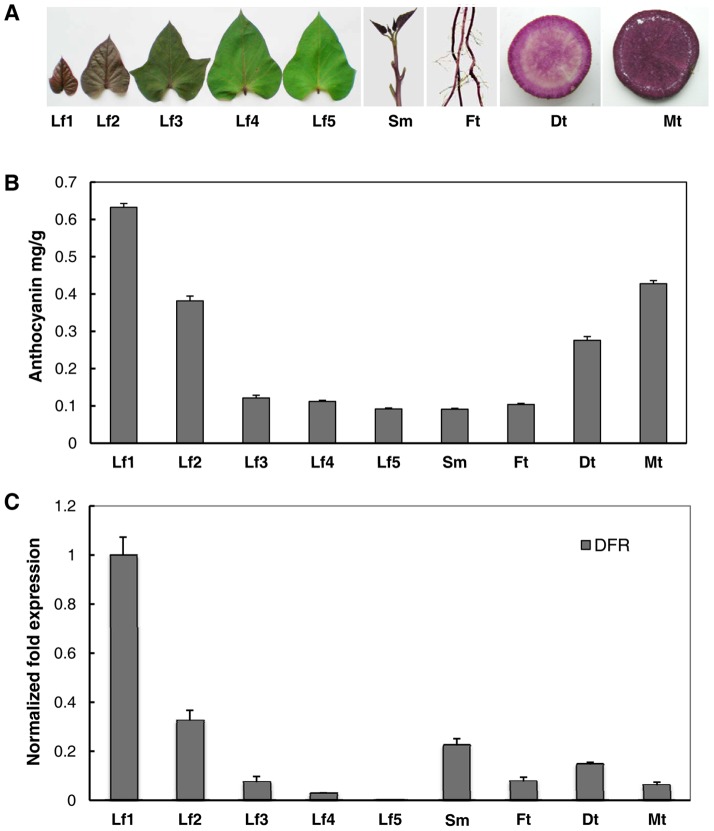
Anthocyanin accumulation and *IbDFR* gene expression in various organs of purple sweet potato cv. Ayamurasaki. A, The phenotypes of the different organs. Lf1, Lf2, Lf3, Lf4 and Lf5 represent the leaves of different developmental stages; Sm, stem; Ft, fibrous root; Dt, developing root; Mt, mature root. B, Different levels of anthocyanin accumulation. C, Expression profile of the *IbDFR* gene in different organs by real-time quantitative polymerase chain reaction. Values represent the mean ± SD (n = 6).

To verify the relationship between *IbDFR* expression and anthocyanin accumulation, the expression profile of *IbDFR* in different tissues was analyzed using real-time qPCR. *IbDFR* transcripts were detected in all tested organs at different expression levels. In leaves, *IbDFR* expression correlates with leaf development, with the most abundant expression in the immature leaf (Lf1) and the least abundant in the fully developed leaf (Lf5) (Figures 2A, 2C). The expression level of Lf1 is 1,000-fold that of Lf5. Lf1 also accumulated the highest level of anthocyanin, followed by the order of Lf2, Lf3, Lf4 and Lf5 (Figures 2B, 2C). Lf1 reached a concentration of 0.6324 mg/g, a 6.89-fold increase over Lf5. Therefore, the expression pattern of *IbDFR* corresponds to the anthocyanin accumulation in leaves. Interestingly, in roots, *IbDFR* is more abundantly expressed in Dt (Figure 2C), although it accumulated higher levels of anthocyanins in Mt (0.4276 mg/g, Figure 2B). In stem, *IbDFR* expression was strongly associated with anthocyanin accumulation. These findings suggest that *IbDFR* expression is associated with anthocyanin biosynthesis activation.

### Complementation of DFR function in Arabidopsis *tt3* mutants

**Figure 3:**
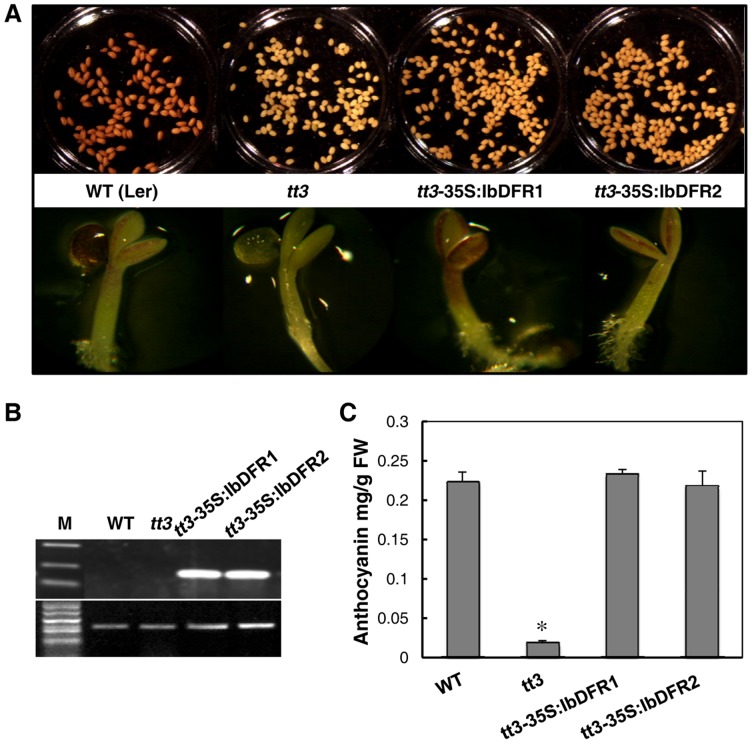
Phenotype complementation in T3 lines of transgenic Arabidopsis *tt3* mutant expressing *IbDFR*. A, Phenotype of the transgenic lines and the wild type (WT, Lerberg) in their seed coats, cotyledons and hypocotyls. B, Expressional analysis of the *IbDFR* gene by reverse transcription polymerase chain reaction in the wild-type, *tt3* mutant and transgenic lines. C, Relative quantitation of anthocyanin contents in the wild-type, *tt3* mutant and transgenic lines. The error bars show standard error of the mean (n = 9). The asterisks indicate a significant difference from that of WT at * P<0.05 by *t*-test.

To validate the *IbDFR* function in anthocyanin biosynthesis, the *IbDFR* gene was introduced and expressed in *Arabidopsis tt3* mutants [22]. The *tt3* mutants failed to accumulate brown tannins in their seed coats and anthocyanin pigments in their cotyledon or hypocotyls (Figure 3A). RT-PCR analysis confirmed the overexpression of *IbDFR* in transgenic lines of Arabidopsis *tt3* mutants (Figure 3B). Phenotypic investigations the T3 homologous transgenic lines showed restoration of the pigmentation of their seed coats and purple coloration in the cotyledons and hypocotyls when their seedlings were grown on medium containing 3% sucrose (Figure 3A). The anthocyanin levels in the cotyledons could be restored as close as 105% (DFR1) and 97.9% (DFR2) in the WT plants (Figure 3C). Taken together these results demonstrate that the purple sweet potato *DFR* gene is fully functional for anthocyanin biosynthesis in Arabidopsis.

### Interference with *IbDFR* gene expression in sweet potato

**Figure 4:**
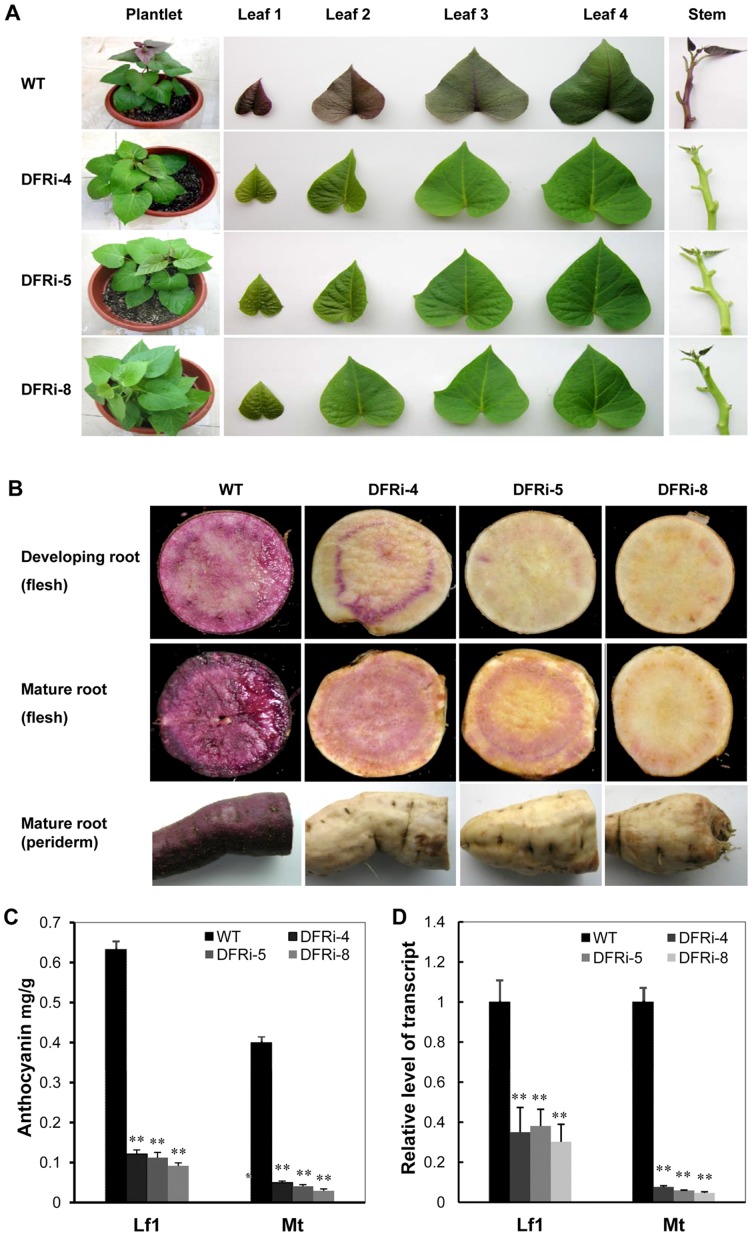
Phenotypic evaluation and anthocyanins measure of wild-type (WT) and DFRi transgenic sweet potato. A, The status of anthocyanin accumulation in shoots of the transgenic plant lines and the WT. B, Anthocyanin accumulation in the developing and mature storage roots of the DFRi transgenic lines and the WT. C, Quantitation of anthocyanin contents in the young leaves (Lf1) and mature roots (Mt) of the DFRi transgenic plants compared with those of the WT. D, Real-time reverse transcription polymerase chain reaction analysis of *IbDFR* expression in the Lf1 and Mt of the DFRi transgenic plants and the WT plants. The actin gene was used as an internal control. Values represent the mean ± SD (n = 6) Asterisks indicate a significant difference from that of the WT at ** P<0.01 (t-test).

Transgenic sweet potato transformed by the binary vector pRNAi-DFR (DFRi) was confirmed by Southern blot analysis using the *Eco*RI-digested genomic DNA hybridized with digoxigenin-labeled hygromycin phosphotransferase gene (*hpt*, Figure S2B). The number of integrated transgenes in the DFRi lines ranged from one to two among five tested lines (Figure S2B) which showed the absence of anthocyanin accumulation in their shoots. The intensity of the typical purple color in the young leaves and stems was dramatically reduced in the transgenic plants compared to the WT plants (Figure 4A). The accumulation of purple pigments was inhibited in both the periderm and in the flesh of the transgenic storage roots (Figure 4B). Their fleshes are light yellow with dispersed light purple coloration. Quantification of anthocyanins extracted from young leaves and storage roots of transgenic plants showed decreased anthocyanin accumulation (Figure 4C). DFRi-8 only reached 0.09174 mg/g in young leaves and 0.02918 mg/g in mature roots compared with 0.6324 mg/g and 0.3391 mg/g of the WT. Anthocyanin content in young leaves of the WT is 5× greater than those of transgenic DFRi-4 plants. Slight variation of anthocyanin levels was detected among the three transgenic lines (Figure 4C). The quantification of anthocyanin content is in agreement with the phenotype changes in their young leaves and storage roots (Figures 4A-C).

### Expression level of *IbDFR* gene in transgenic DFRi sweet potato

The expression levels of the endogenous *IbDFR* gene in the young leaves and storage roots of transgenic sweet potato plants were examined by real-time PCR analysis. In young leaves (Lf1), the *IbDFR* expression levels in the WT were 2.85-fold, 2.63-fold and 3.33-fold those of DFRi-4, DFRi-5 and DFRi-8, respectively (Figure 4D). Dramatically lower expressions of *IbDFR* were detected in the mature storage roots of DFRi-4, DFRi-5 and DFRi-8 than that of the WT. More than a 12-fold difference was detected between the WT and transgenic plants (Figure 4D), and the suppression was more efficient in the mature storage roots than leaves in these transgenic plants. These results showed inhibited *IbDFR* transcription in transgenic plants.

### Changes in other flavonoids and antioxidant capacity

**Figure 5:**
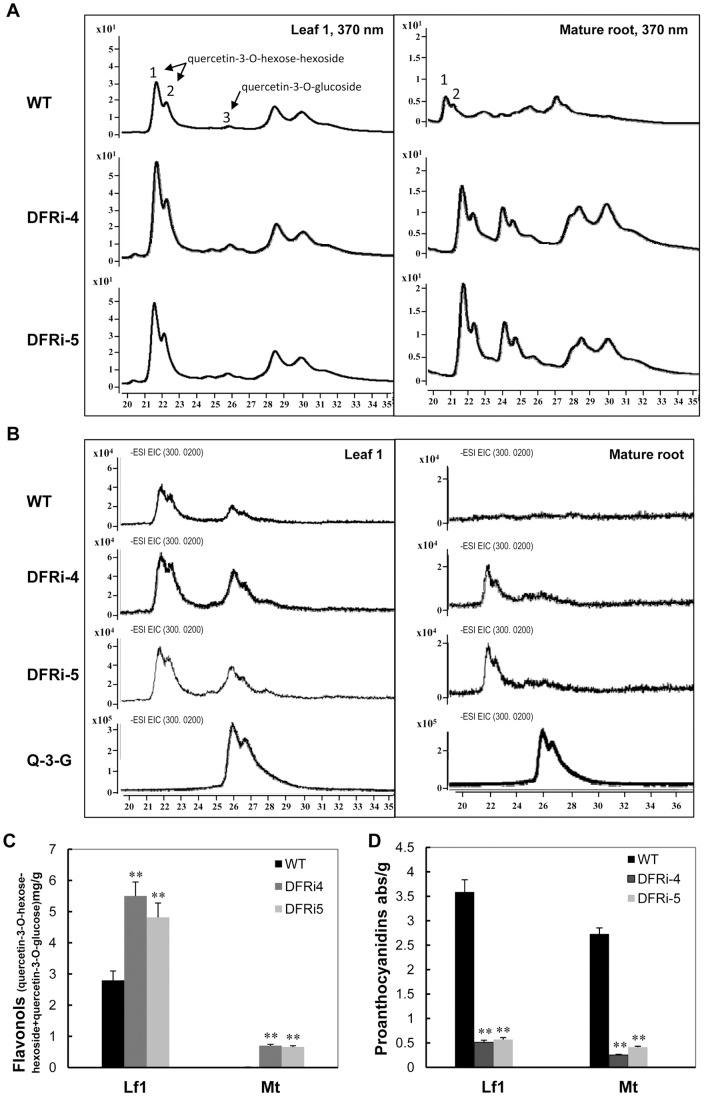
HPLC-MS analyses of flavonols and quantification of proanthocyanidin in wild-type (WT) and DFRi sweet potato. A, A HPLC chromatograms of the samples from young leaves (Leaf 1) and mature roots of DFRi and WT plants at 370 nm. B, Monitored ions with an *m*/*z*-value of 300.02 (molecular weight of quercetin) in the young leaves and mature roots of the DFRi and WT plants; Quercetin-3-O-glucoside (Q-3-G) was used as a standard. C, Content changes of flavonols (quercetin-3-O-hexose-hexoside and quercetin-3-O-glucoside) in young leaves and mature roots of the DFRi and WT plants. D, Content changes of proanthocyanidin in young leaves and mature roots using the spectrophotometry method. Lf1, leaf 1; Mt, mature root. Values represent the mean ± SD (n = 6). Asterisks indicate a significant difference from that of wild-type (WT) plants at *P<0.05 or **P<0.01 (t-test).

The DFRi lines showing the absence of anthocyanins were detected the higher concentration of flavonols in their leaves and storage roots (Figures 5A-C). Two flavonols, quercetin-3-O-hexose-hexoside ([M-H]^−^625) and quercetin-3-O-hexoside ([M-H] ^−^463), which recently identified in purple sweet potato [50] was illustrated. The peak 1 and 2 of quercetin-3-O-hexose-hexoside were confirmed based on its mass spectra and by the comparisons with the already characterized quercetin-3-O-hexose-hexoside. HPLC-MS detection, at 21.798 min and 22.376 min, between WT and DFRi leaf and root extracts clearly showed that quercetin-3-O-hexose-hexoside was accumulated more in DFRi lines (dominant ions *m*/*z* 625.14, 300.02)(Figures 5A,5B). In DFRi-4, for example, the contents of quercetin-3-O-hexose-hexoside in young leaf and mature root were 4.949 mg/g and 0.672 mg/g, respectively, about 2-fold and 75-fold more than those of WT. Another quercetin-glycosides quercetin-3-O-glucoside (dominant ions *m*/*z* 463.08, 300.02, 26.05min) was detected in leaves and roots according to the standard sample of quercetin-3-O-glucoside (Figures 5A,5B). The DRFi lines also showed higher content of quercetin-3-O-glucoside in their leaves and roots than those of WT. Totally, the flavonol content (quercetin-3-O-hexose-hexoside + quercetin-3-O-glucoside) in the leaf and storage root of DFRi-4 were 5.500 mg/g and 0.701 mg/g, respectively, which are about 2-fold and 70-fold higher than those of WT, showing an increased biosynthesis of flavonol in the DFRi plants. Similar changes were made in the DFRi-5 plant (Figure 5C). Therefore, compared to wild type, an increase of the total flavonols in DFRi was also observed.

The content of proanthocyanidin was also detected by spectrophotometry. At 500-nm absorbance, the transgenic plants displayed dramatically reduced proanthocyanidin content. For example, the DFRi-5 plant had values of 0.569 abs/g and 0.413 abs/g in the leaf and storage root, respectively, which were reduced by 6.3- and 6.6-fold those of the WT (Figure 5D).

**Figure 6:**
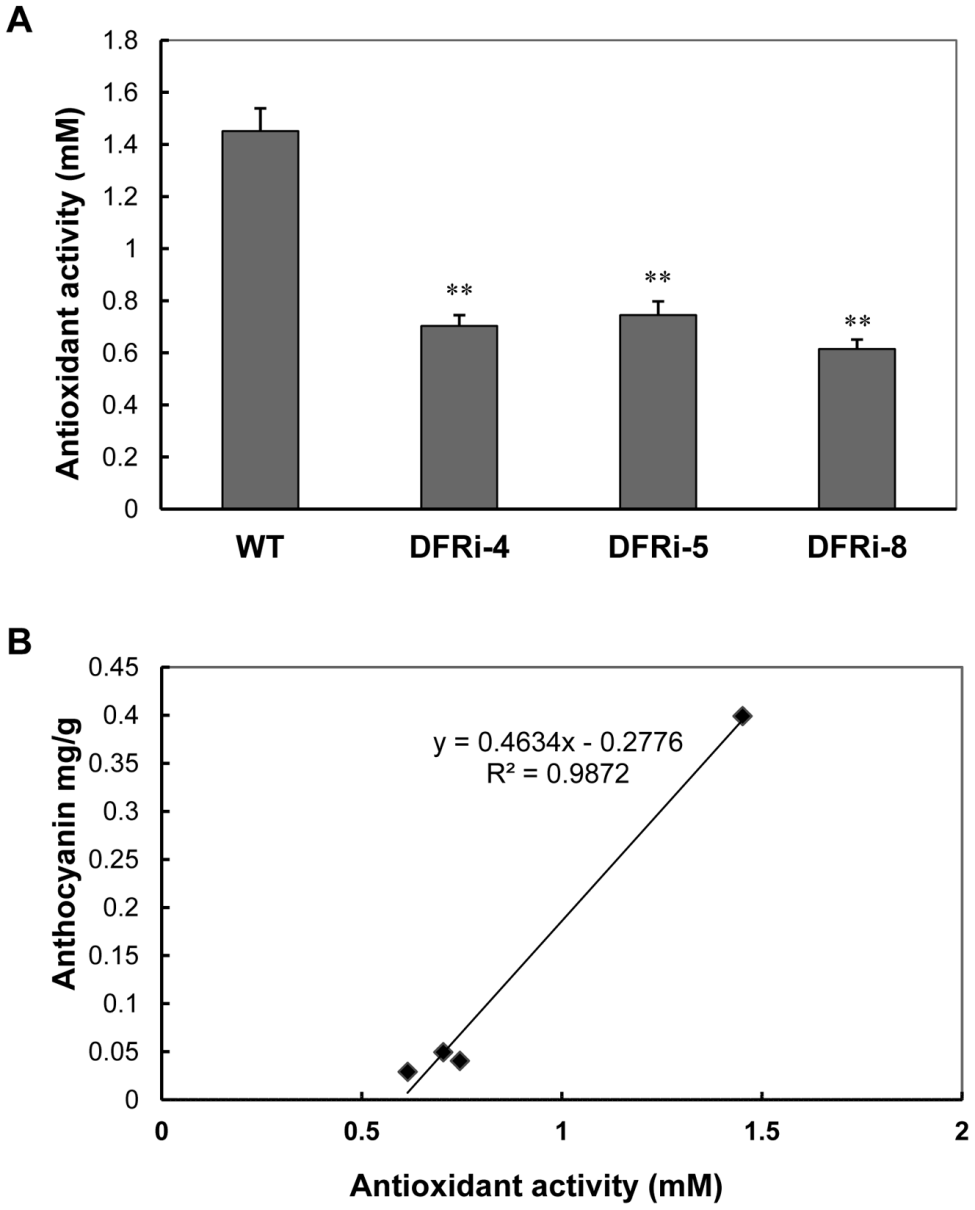
Comparison of antioxidant activity in wild-type (WT) and transgenic lines. A, Antioxidant capacity in the mature storage roots of the transgenic lines and the WT measured by the FRAP assays. Values represent the mean ± SD (n = 6). Asterisks indicate a significant difference from that of WT at * P<0.05 or ** P<0.01 (*t*-test). B, Linear correlation (R^2^ = 0.9872) between antioxidant activity and anthocyanin content in sweet potato.

FRAP assays were conducted to further test the antioxidant activity of the transgenic lines (Figure 6). Purple storage roots of the WT contain significantly higher antioxidant capacities than those of the DFRi transgenic plants, which have the cream-colored storage roots (Figure 4B). The antioxidant activities of DFRi-4, DFRi-5 and DFRi-8 remained at 0.703 mM, 0.745 mM and 0.614 mM, respectively, which was significantly lower than that of the WT 1.451 mM (Figure 6A). In general, the relationship between antioxidant capacity and anthocyanin concentration in sweet potato is strongly correlated (R^2^ = 0.9872; Figure 6B). This finding indicates that anthocyanins are major contributors of antioxidants in sweet potato.

### Impact on cold tolerance of DFRi transgenic plants

**Figure 7:**
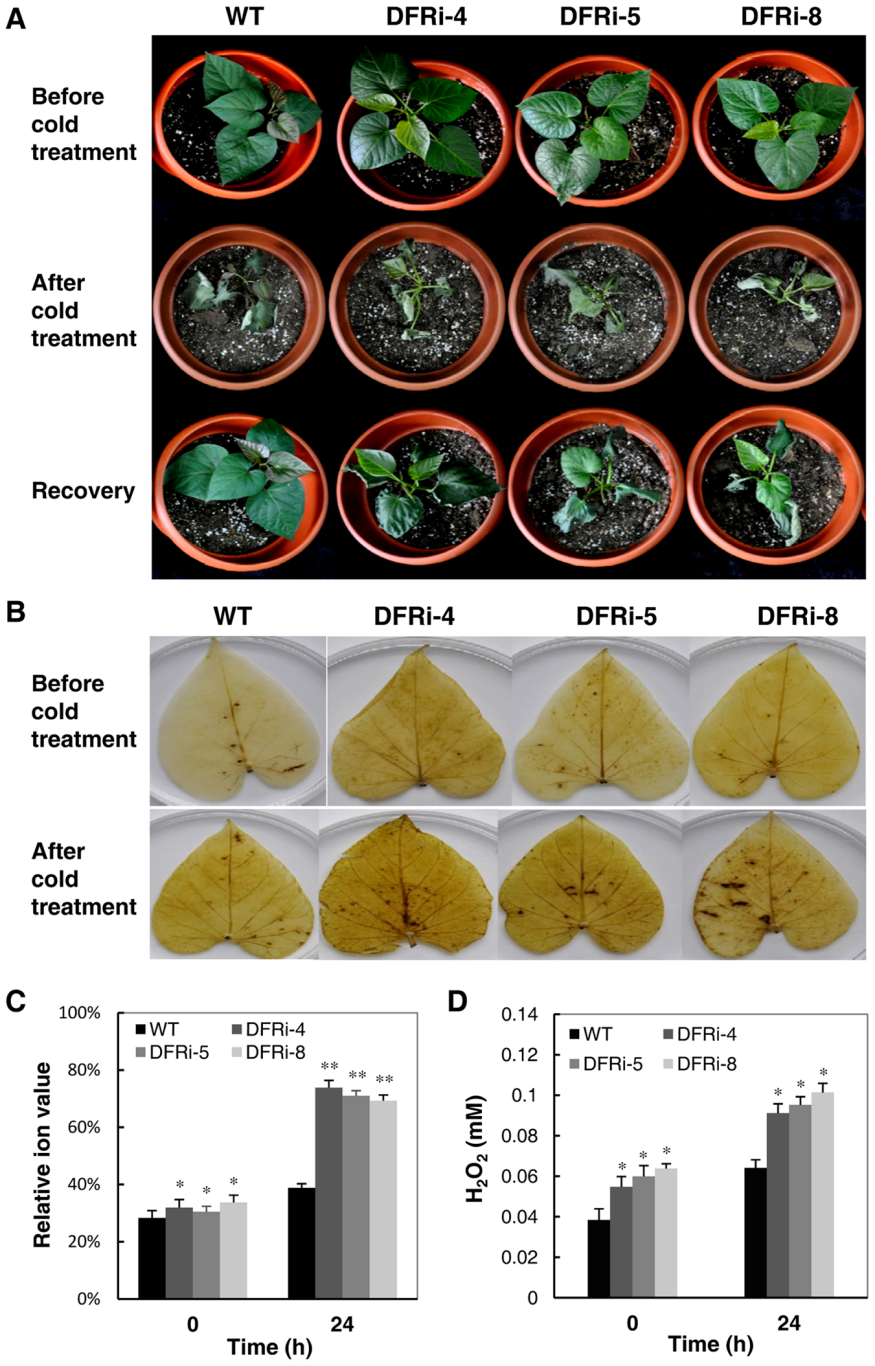
Phenotypic and physiological analyses of the wild-type (WT) and DFRi transgenic plants under cold treatment. A, Phenotypic changes before (upper panel) and after (middle panel) cold treatment (4°C for 24 h) and the recovery status (25°C for 2 h, lower panel). B, Visualization of cold-induced H_2_O_2_ production by 3-diaminobenzidine staining in the leaves of RNAi and WT plants. C, Ion leakage in the cold-treated leaf discs of DRFi transgenic plants compared to the WT plants. D, H_2_O_2_ contents in the leaves of transgenic and WT plants after cold treatment. D, Values represent the mean ± SD (n = 6). Asterisks indicate a significant difference from that of the WT at * P<0.05 or ** P<0.01 (*t*-test).

When exposed to cold for 24 h, the leaves of WT and DFRi plants all showed severe wilting and curling, indicating stress-induced damage. Upon recovering for 2 h at 25°C, the WT plants had almost fully recovered to the initial phenotype prior to treatment, whereas the DFRi plants still exhibited leaf wilting (Figure 7A). In addition to phenotypic changes, the electrolyte leakage levels were also increased in the transgenic lines and WT after cold treatment (Figure 7C). Before treatment, the electrolyte leakage rates of the DFRi plants were 31.9%, 30.4% and 28.3%, respectively, and did not differ significantly from those of the WT (28%). After 24 h of cold stress treatment, the electrolyte leakage rates of DFRi-4, DFRi-5 and DFRi-8 reached 73.8%, 70.9% and 69.3%, respectively, whereas that in the WT reached only 38.7%. The electrolyte leakage rates in the transgenic lines increased more than 2-fold, while that of the WT increased only 1.36-fold (Figure 7C).

To determine whether anthocyanin is involved in scavenging excess ROS in sweet potato, H_2_O_2_ content was measured before and after the cold treatment. Generally, the H_2_O_2_ contents in the leaves of WT and transgenic lines were increased during cold treatment (Figures 8B,8D), indicating an enhanced ROS turnover during cold stress. After 24 h, all transgenic lines accumulated significantly more H_2_O_2_ in their leaves than the WT (Figure 7D). For example, the change of H_2_O_2_ content in the DFRi-8 sample was from 63.8 µM to 101 µM, a 37.2-µM increase; for the WT, from 38.3 µM to 64.1 µM, only a 25.8 µM increase. Obviously, there was an increase in ROS accumulation in the transgenic plants compared with the WT. Thus, anthocyanins show antioxidant properties in sweet potato *in vivo* and protect the plants from oxidative damage under abiotic stress by increasing ROS scavenging.

## Discussion

In flavonoid biosynthesis, DFR is a key enzyme in the catalysis of the stereospecific reduction of dihydroflavonols to leucoanthocyanidins that uses NADPH as a cofactor. It has been reported that anthocyanin concentration and DFR enzymatic activity are strongly correlated with flower pigmentation and during fruit ripening [51], [52]. Although the sequences of DFRs in sweet potato had been reported [53], the function of DFR in purple sweet potato has been less addressed and remains largely unknown. In this report, the expression pattern and function of *IbDFR* in purple sweet potato were intensively studied. *IbDFR* expression was strongly associated with anthocyanin accumulation in different organs and development, which suggests that *IbDFR* expression is a key step in anthocyanin biosynthesis (Figure 2). As evidenced in the DFRi transgenic plants, the accumulation of purple pigment in their leaves, stems and storage roots was dramatically reduced (Figures 4A-C). Furthermore, the pigmentation phenotype in *Arabidopsis* was fully complemented in the *tt3* mutants that express the *IbDFR* gene (Figure 3). When the anthocyanin biosynthesis branch was blocked, the proanthocyanidin metabolites decreased but flavonol biosynthesis increased, as evidenced by the increase of quercetin-3-O-hexose-hexoside and quercetin-3-O-glucoside significantly (Figure 5). These results suggest that the change of *IbDFR* gene expression affects the flavonoid flux in purple sweet potato.

Anthocyanins and flavonols share common precursors, dihydroflavonols, which are substrates for both flavonol synthase and dihydroflavonol 4-reductase. Two quercetin derivatives were identified and characterized as the major flavonols in the DFRi and WT plants (Figures 5A-C), which are consistent with a previous report of two major flavonols of sweet potato [50]. The increased accumulation of the two flavonol components in the DFRi plants indicates that, when the anthocyanin accumulation is inhibited, the increased conversion of dihydrokaempferol to quercetin by FLS and production of flavonols might occur. It has been reported that inactivation of DFR increases accumulation of quercetin in *Arabidopsis* [54], [55], whereas *fls1* mutants have elevated anthocyanin content [56], [57]. Our results agree with these findings and indicate that metabolic flux in the flavonoid biosynthetic pathway is controlled by substrate competition between FLS and DFR. It is likely that the change of FLS-to-DFR ratio in DFRi plant causes the increased accumulation of flavonols.

The redirection of the metabolic flux toward flavonols through downregulation of *DFR* offers a practical approach to the metabolic engineering of plant flavonoids. Indeed, under different biotic and abiotic stress, the fluctuation between flavonols and anthocyanins had been observed in many plants [58], [59], [60]. This reflects that, as an integrative pathway, the metabolic flux of anthocyanin and other flavonoid biosynthetic pathways can be affected by environmental factors, leading to an alternative channel. The increase of the major flavonols, quercetin-3-O-hexose-hexoside and quercetin-3-O-glucoside, in DFRi transgenic sweet potato (Figure 5C) might also suggest that blocking the anthocyanin biosynthesis causes the increase of flavonols possibly by increased substrates.

Using the WT and transgenic sweet potato, the ROS scavenging capacity of anthocyanins was validated *in vivo* with or without stressful conditions (Figure 7), although a few studies have shown a similar effect using extracted anthocyanins from various sweet potato genotypes [35], [61], [62]. The reduced antioxidant activity in sweet potato lacking anthocyanins also confirmed the protective function of anthocyanins in plants as a conserved mechanism of stress resistance.

It has reported that anthocyanin-rich sweet potato could enhance the expression of antioxidant enzymes, such as SOD, CAT and GPX, in rat livers [35]. The complexity of nutritional components in sweet potato creates uncertainty in the direct evaluation of anthocyanin functions as reported by Davies [63]. Use of the WT and DFRi transgenic sweet potato plants will provide suitable material for various studies under the same genetic background except for the anthocyanin pathways. The reduced antioxidant capacity and greater sensitivity to cold treatment of the DFRi transgenic plants compared to the WT plants implies the important biological role of anthocyanins against oxidative stresses *in vivo* (Figures 6,7). Plants under abiotic stress have evolved a defense system against oxidative stress by increasing the activity of ROS scavenging enzymes [64]. Many investigators have demonstrated that purple sweet potato could improve the antioxidative activity within cells because of its strong free radical scavenging activity [62], [65], [66]. Abiotic stresses such as low temperature, which is known to disturb redox homeostasis in plant cells, could induce ROS production, resulting in oxidative stress. ROS have been implicated in all stress types, and if they are not scavenged sufficiently, programmed cell death might occur [67]. In our study, oxidative stress was enhanced in the cold-stressed leaves of the sweet potato as reflected by the increased H_2_O_2_ content and ion leakage; however, these stress levels were much higher in the transgenic plants than in the WT plants (Figure 7). These observations strongly support the notion that anthocyanins participate in maintaining ROS homeostasis during the plant development and growth, a finding that is in agreement with those of earlier reports [68]. Thus, improvement of stress tolerance by enriching anthocyanin production in plants is considered an alternative approach to the nutritional point of view in crop improvement.

In conclusion, this study validated the biological function of the *IbDFR* gene in anthocyanin biosynthesis. The changes of anthocyanin accumulation by the downregulation of *IbDFR* expression also influence the flux distribution of other flavonoids, such as flavonols and proanthocyanidins, in purple sweet potato. We also verified the antioxidant property of anthocyanins in sweet potato, mainly through the induction or activation of ROS scavenging. Thus, this study has increased our understanding of DFR function in flavonoid metabolism, although some questions, such as how anthocyanin improves the ROS-scavenging capacity, remain to be answered. Nevertheless, the purple sweet potato and its transgenic plants provide invaluable sources of information for nutrition studies.

## Supporting Information

Figure S1 **Simplified scheme of the anthocyanin biosynthesis pathway.** PAL, phenylalanine ammonialyase, C4H, cinnamate 4-hydroxylase, 4CL, 4-coumarate CoA ligase, CHS, chalcone synthase, CHI, chalcone isomerase, F3H, flavanone 3-hydoxylase, DFR, dihydroflavonol 4-reductase, ANS, anthocyanidin synthase, GT, anthocyanin glucoyltransferase, FNS, flavone synthase, IFS, isoflavone synthase, FLS, flavonol synthase, LAR, leucoanthocyanidin reductase, ANR, anthocyanidin reductase.
(TIF)

Figure S2 **Schematic representation of expression cassette and Southern blot analysis of transgenic sweet potato.** A, Schematic representation of the hairpin double-stranded RNA expression cassette in the T-DNA region of the pRNAiDFR vector; B, Southern blot analysis of wild-type (WT) and transgenic plants using the DIG-labeled *hpt* partial gene as a probe.
(TIF)
