# Supplementary figures and images for: Functional Characterization of Dihydroflavonol-4-Reductase in Anthocyanin Biosynthesis of Purple Sweet Potato Underlies the Direct Evidence of Anthocyanins Function against Abiotic Stresses
Source: PLoS One. 2013 Nov 4;8(11):e78484. doi: 10.1371/journal.pone.0078484 (PMC3817210; doi:10.1371/journal.pone.0078484)

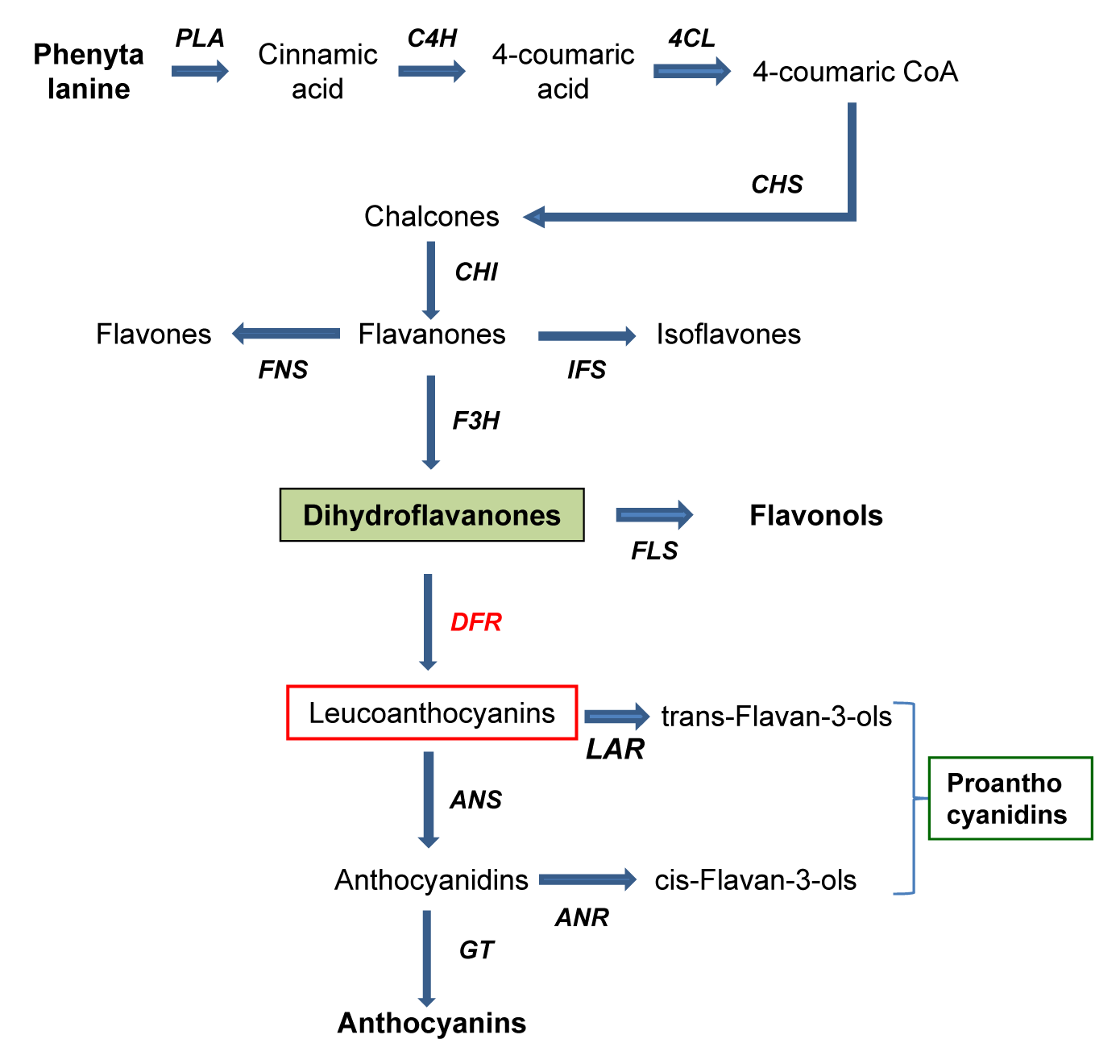

Supplement: Figure S1 — Simplified scheme of the anthocyanin biosynthesis pathway. PAL, phenylalanine ammonialyase, C4H, cinnamate 4-hydroxylase, 4CL, 4-coumarate CoA ligase, CHS, chalcone synthase, CHI, chalcone isomerase, F3H, flavanone 3-hydoxylase, DFR, dihydroflavonol 4-reductase, ANS, anthocyanidin synthase, GT, anthocyanin glucoyltransferase, FNS, flavone synthase, IFS, isoflavone synthase, FLS, flavonol synthase, LAR, leucoanthocyanidin reductase, ANR, anthocyanidin reductase. (TIF) [file pone.0078484.s001.tif]

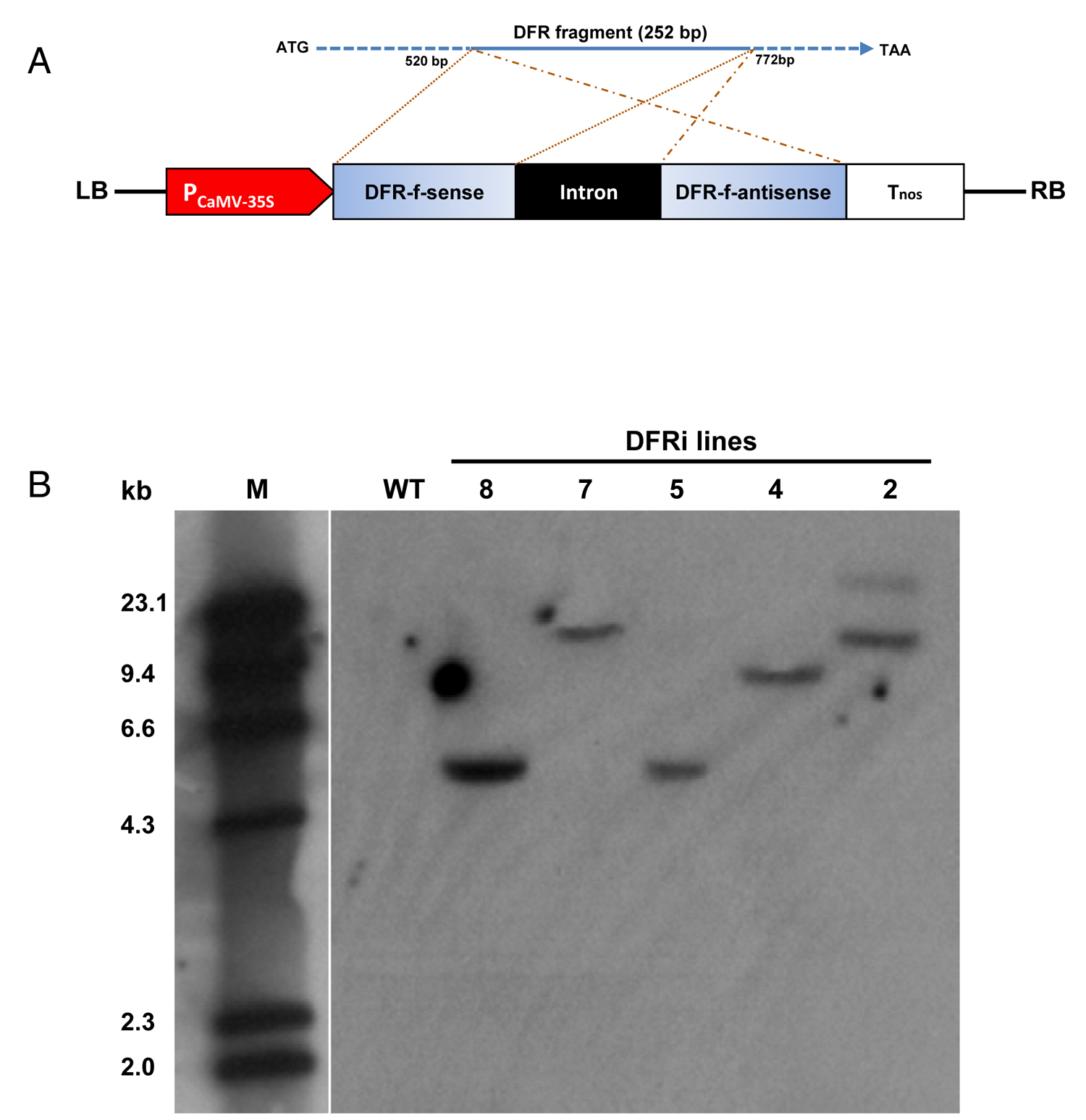

Supplement: Figure S2 — Schematic representation of expression cassette and Southern blot analysis of transgenic sweet potato. A, Schematic representation of the hairpin double-stranded RNA expression cassette in the T-DNA region of the pRNAiDFR vector; B, Southern blot analysis of wild-type (WT) and transgenic plants using the DIG-labeled hpt partial gene as a probe. (TIF) [file pone.0078484.s002.tif]
